# Impacts of land use and land cover change on ecosystem service values in the Afroalpine area of Guna Mountain, Northwest Ethiopia

**DOI:** 10.1016/j.heliyon.2022.e12246

**Published:** 2022-12-09

**Authors:** Tatek Belay, Tadele Melese, Abebe Senamaw

**Affiliations:** a Department of Geography and Environmental Studies, College of Social Science and Humanities, Debre Tabor University, P. O. Box 272, Debre Tabor, Ethiopia; b Department of Natural Resource Management, College of Agriculture and Environmental Science, Bahir Dar University, P. O. Box 5501, Bahir Dar, Ethiopia

**Keywords:** Ecosystem service value, Google earth engine, Guna mountain, Land use land cover

## Abstract

Ecosystem service changes caused by land use and land cover change (LULCC) is an important indictor and early warning of ecological changes. However, few attempts have been made to evaluate the effects of LULCC on ecosystem services in the Afroalpine highlands of Northwestern Ethiopia. Therefore, this study aimed to analyze the impacts of LULCC on ecosystem services values in the afro-alpine area of Guna Mountain, Northwestern Ethiopia. Image classification was carried out using Landsat imageries of 1995, 2008, and 2020 following Random Forest algorithm with Google Earth Engine(GEE) based on ﬁltered sample points. A modified benefit transfer method was used to evaluate ecosystem service value (ESV) changes in response to LULCC. The results revealed that the most notable feature of LULCC in the afro-alpine area of Guna Mountain was the expansion of cropland and built-up areas at the expense of grassland, forest, and shrubland. The overall ESV of the study site was estimated at USD 46.97 × 10^6^ in 1995, USD 36.77 × 10^6^ in 2008, and USD 37.19 × 10^6^ in 2020. The net ESVs of the study site declined by USD 9.78 × 10^6^ between 1995 and 2020. The regulating service values accounted for the greatest share, accounting for over 42% in all periods, followed by provisioning and supporting service values, which accounted for over 29% and 13%, respectively, while cultural services accounted for the smallest amount of the total ESV. The ecosystem service value of food production experienced the highest increase. However, the values of the remaining 16 types of ecosystem services declined with varying degrees of reduction over the study periods. The results of this study is necessary for land-use planners and decision-makers who require site-specific information on impacts of LULCC on ecosystem service.

## 1 Introduction

Land use and land cover (LULC) are frequently used interchangeably, but they have very different meanings. Land use relates to the function of the land, such as recreation, agriculture, wildlife habitat, and so on, whereas land cover refers to the ground's surface cover, such as water, vegetation, bare soil, and so on (Fisher et al., 2005; Lambin et al., 2003). As Fuladlu (2022) pointed out land use refers to human utilization of the land while land cover refers the physical and biological condition of the land. The earth's surface land use and land cover (hereinafter LULC) has been changing rapidly (Goldewijk and Ramankutty, 2004). In Africa, the rates and intensities of LULC dynamics are changing drastically, which has significant consequences for changes in ecosystem service (Arowolo et al., 2018; Badamfirooz and Mousazadeh, 2019), which contributes to household food insecurity.

Ecosystems offer a variety of valuable goods and services that support both nature and human well-being (MEA, 2005), which is governed by the underlying ecological processes. Ecosystem services collectively refer to all ecosystem products and services (Daily et al., 2000). Ecosystem services formed and kept by the ecosystem provide crucial roles of provision (e.g., food, fresh water, and fuel), regulation (e.g., climate regulation, soil, water, and pollination), cultural (e.g., recreation, cultural heritage, and inspirational value), and other services (e.g., production and nutrient cycle) (MEA, 2005; Xie et al., 2015). Many of these services are essential for life on earth and the integrity of ecosystems (Yan et al., 2017).

Despite the outstanding contribution of ecosystem services to support nature and human well-being, human activities can alter ecological environments and natural ecosystems by changing the structure and pattern of LULC (Belay and Mengistu, 2019; Lambert et al., 2017). LULC are the main determinants of most landscapes' structure, functions, and dynamics worldwide (Liu et al., 2020). It is a crucial way human activities aﬀect ecosystems (Li et al., 2018). Over recent decades, LULC has been substantially changed (Belay and Mengistu, 2019; Betru et al., 2019; Habte et al., 2021; Sisay et al., 2021), which have had an incredible impact on ecosystem services by influencing ecosystem patterns and processes (Gashaw et al., 2018; Kindu et al., 2016). Humans benefit directly from changing land use types but dramatically alter ecosystem services. Changing land use types provides immediate economic benefits for humans but dramatically alters or causes imbalances in ecosystem services. These changes exemplify air quality, water resources, disaster prevention, and other negative aspects of human health. Costanza et al. (2014) stated that LULC changes decreased the entire monetary value of ecosystem services. Globally by 4.3–20.2 trillion USD per year between 1997 and 2011. The consequences of LULC change on ecosystem services contrast over time and place (Brander et al., 2012; Bryan, 2013; de Groot et al., 2012; Song and Deng, 2017; Tolessa et al., 2017).

Many studies have been carried out all over the world to assess the change in ecosystem service valuation (ESV) caused by LULC changes at a local scale (e.g., Brander et al., 2012; Celentano et al., 2017; Gashaw et al., 2018; Kindu et al., 2016; Song and Deng, 2017; Sun et al., 2021; Tolessa et al., 2017; Woldeyohannes et al., 2020). Studies showed that ecosystems provide unique services with varying quality and quantity depending on the type and circumstances of LULC. For example, a forest was quite different in service provision than grassland or aquatic ecosystem (Costanza et al., 1997). Each LULC type provides a unique service that others can not replace (Celentano et al., 2017). Compared to degraded forests, intact forests provide various ecosystem services.

Some studies showed (e.g., Balzan et al., 2018; MEA, 2005; Narducci et al., 2019) that the expansion of built-up areas reduces the ecosystem's biodiversity and resilience, reducing its overall potential to provide ESV. However, the economic benefits of expanding the built-up area are also substantial (Díaz et al., 2015). Hence, preventing the expansion of built-up areas through unforesightful policies could negatively impact economic growth (Bertinelli and Black, 2004). Overall, ecosystem functioning and resilience may be affected due to LULC change and threaten ecosystems' ability to provide services for current and upcoming generations. These threats emphasize the importance of quantifying and evaluating the impact of LULC changes on ecosystem services (Hasan et al., 2020; Zhang et al., 2019).

Several studies have been conducted on LULC changes in Ethiopia. They have primarily focused on the dynamics of cover changes and associated causes (Belay and Mengistu, 2019; Betru et al., 2019; Sisay et al., 2021), with little emphasis on the implications of LULC changes on ecosystem services (Gashaw et al., 2018; Kindu et al., 2016; Tolessa et al., 2017). Changes in LULC can lead to changes in ecosystem services because ecosystem service provision is directly related to the type of ecosystem (Aziz, 2021; Hasan et al., 2020; Sun et al., 2021; Yee et al., 2021). As a result, unprecedented LULC changes due to human activities lead to the degradation of ecosystem services, compromising their potential to provide services (Alcamo et al., 2005; MEA, 2005), which may affect the well-being of the upcoming generation and other services. Accordingly, LULC change has been recognized as one of the significant drivers of ecosystem service loss (Costanza et al., 2014; Tekalign et al., 2018; Tscharntke et al., 2005).

The Ethiopia highlands have been under intense pressure due to their favorable climatic situations for agricultural, human, and animal health compared to the lowlands. This results in environmental resource deterioration, mainly LULC changes (Hurni et al., 2010; Lemenih and Teketay, 2005; Tolessa et al., 2016), which causes a reduction in ecosystem services (Kindu et al., 2016). LULC changes are also caused by expansion of cultivation, urbanization, deforestation, drought, and insufficient land-use planning resulting in considerable losses in ecosystem services (Gashaw et al., 2018; Kindu et al., 2016; Shiferaw et al., 2019).

The afro-alpine area of Guna mountain is one of the most environmentally vulnerable regions in the highland parts of Ethiopia. Recent LULC changes (e.g., Birhanu et al., 2021), where agricultural activities and settlements predominate rural areas and impact ecosystem services.

Data on LULC change and ecosystem service value can be used to find landscape areas most susceptible to alterations in ecosystem services and as a starting point for future land management choices (Martínez-Sastre et al., 2017). Furthermore, ESV studies will help policymakers to consider the trade-off that occurs with LULC change and ecosystem services provision (Costanza et al., 2014; de Groot et al., 2012). However, research on the quantifiable effect of LUCC on the provision of ecosystem service values in afro-alpine areas is inadequate (Solomon et al., 2019).

The afro-alpine environment of Guna mountain is home to nationally and globally significant species and the source of numerous rivers that drains into the three basins of Abay, Tekeze, and Lake Tana. Mount Guna is also the source of the Rib, Gumara, and numerous other rivers that flow into the Lake Tana sub-basin. Millions of the nearby residents depend on the area and its resources for their living. However, population growth and accompanying LULC changes in the site lead to ecosystem degradation and affect the local community that relies on ecosystem service. Its impact could also affect the Abay river basin of Ethiopia. These all indicate the need for immediate development intervention to create a sustainable ecosystem through proper land use planning and management.

To make land-use planning strategies more convenient and sustainable (Martínez-Sastre et al., 2017), understanding ecosystem service dynamics in an ecosystem experiencing LULC change is the first step (de Groot et al., 2012). Furthermore, stating ecosystem services in monetary terms is becoming more prevalent to raise consciousness among users, provide confirmation for decision/policymakers, determine the opportunity costs of rehabilitation, and facilitate payment for ecosystem services (Costanza et al., 2014; de Groot et al., 2012).

Consequently, how LULC changes can disturb ecosystem service values in the Afro-alpine area of Guna mountain can be used to link the importance and benefits of proper land management to land planners, policymakers, and managers. Specifically, this study addresses the following objectives.

- To assess the spatial and temporal dynamics patterns of LUCC in the study area.
- To analyze and predicate the dynamic changes of ESV caused by LULC impacts on the Afroalpine site of Guna mountain in the study period (1995–2020).

Therefore, this will provide a basis for developing proper land-use planning and regulation framework to preserve the sustainability and well-being of the ecosystem and the environment in the Afroalpine area of Guna mountain. Furthermore, the study will help for the achievement of countrywide sustainable development goals (SDGs) associated with food and water security (SDGs 2 and 6) as well as life on land (SDG15) (Katila et al., 2019).

## 2 Materials and methods

### 2.1 Description of the study site

**Figure 1:**
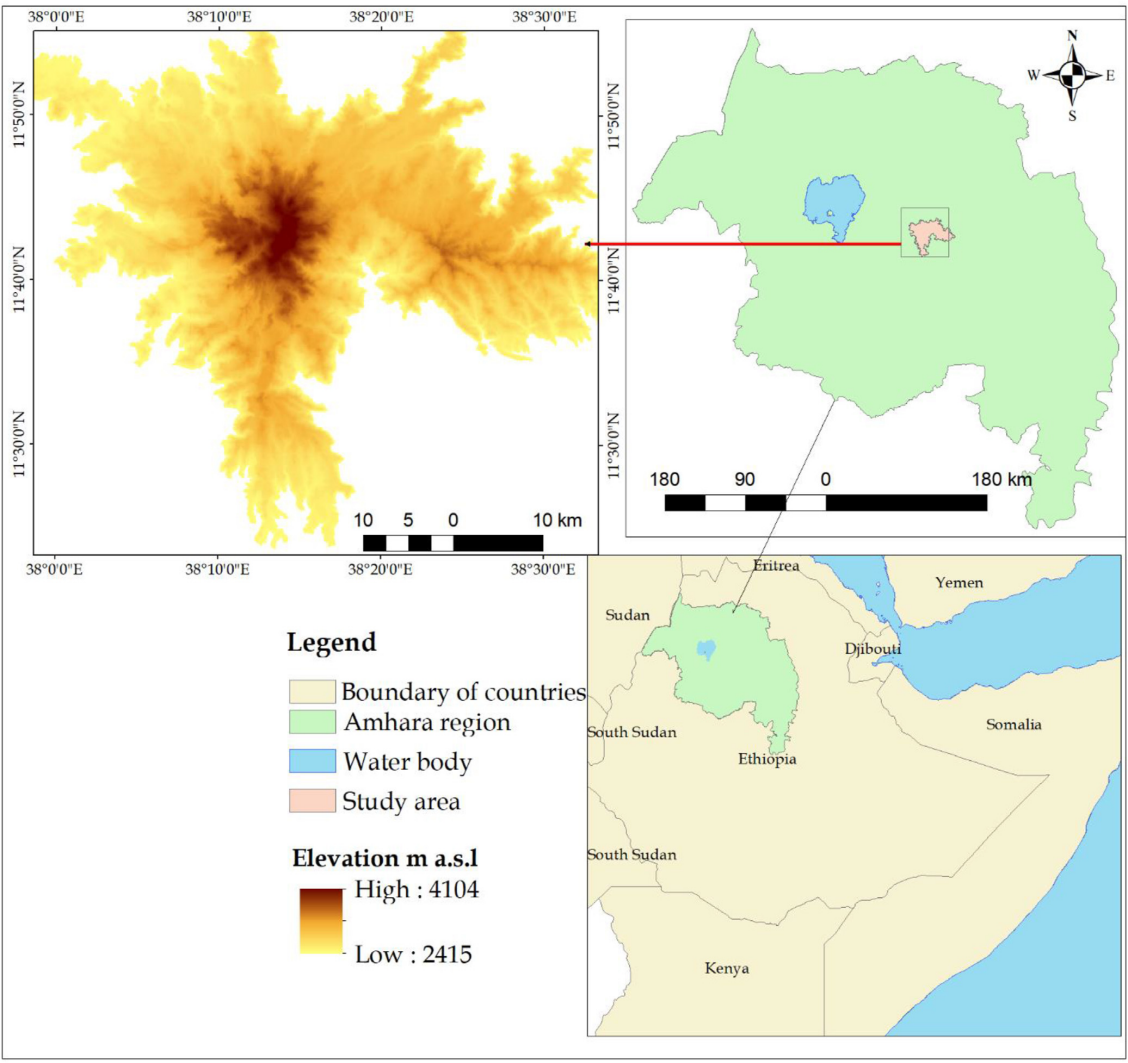
Map of the study area.

The study area, the Afroalpine area of Guna mountain, is in the Abay river basin of Northwestern Ethiopia. Geographically, it is located between 11° 29′ 30″ to 11° 50′ 30″N and 38° 4′ 30″ to 38° 32′ 0″ E (Figure 1). It covers an area of 944.2 km^2^ with an elevation of 2800-4,100 masl. It is located within seven South Gonder zone districts, namely, Simada, Estie, Lay Gayint, Tach Gayint, Guna Begemidir, Sedie Muja, and Farta.

The Guna mountain habitat would preserve 30 higher and small mammals, 139 birds, and about 96 plant species. Furthermore, it is home to six and thirteen endemic animals and birds, respectively (Abebaw, 2016). Numerous streams and rivers that rise from Mount Guna are essential for drinking and agriculture in downstream areas. The mountain is also a major water source for Lake Tana.

The population density is 156.52 people per km^2^. The study area is characterized by undulating landscapes with high interannual climate variability, complicated topography, and acute land pressure are found because of the growing population (CSA, 2007). Subsistence agriculture, predominantly crop farming and animal husbandry, is the primary income source for the study area residents (Birhanu et al., 2021). The ecosystem elements of Mount Guna are vital for the local community's subsistence; they provide food, water, construction materials, fuelwood, etc. Barley and potato are common crops up to 3700 m above sea level. The most common livestock in the study area are horses, cattle, and sheep.

The main soil types in the areas are Cambisols, Leptosols, Luvisols, and Vertisols (Nachtergaele et al., 2012). The topographic climate gradient enables the cultivation of crops of both tropical and temperate origin.

### 2.2 Methods

#### 2.2.1 Data collection

**Figure 2:**
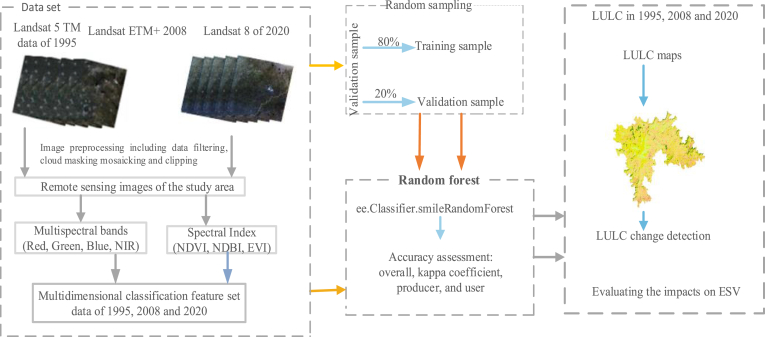
Flowchart of this study methodology (land use land cover (LULC); ecosystem service value (ESV); Near Infrared (NIR); Normalized Difference Vegetation Index (NDVI); Normal Difference Built-up Index (NDBI); Enhanced Vegetation Index (EVI).

The study used multi-temporal data of three periods obtained from Landsat images. The data processing flowchart for this study is presented in Figure 2. It shows the research steps of the LULC classiﬁcation method and change detection. Google Earth Engine (GEE) was used to compute the cloudless composite Landsat images for 1995, 2008, and 2020 (Huang et al., 2017).

#### 2.2.2 Landsat archive data in the Google Earth engine

Google Earth Engine allows users to access archived Landsat data online, including Landsat 5 TM from 1985 to 2011, Landsat 7 ETM+ from 1999 to 2014, and Landsat 8 OLI/TIRS from 2013 to 2021. With a spatial resolution of 30 m, the original images were obtained from the United States Geological Survey (USGS) earth explorer website. The dates for LULC processing were determined by the accessibility of images and critical dates in the transformation of government policies associated with land-related resources.

The images were not taken with the atmospheric correction from USGS by GEE. As a result, cloud mosaic and multi-year image synthesis methods were used to classify Landsat top-of-atmosphere (TOA) reflectance products from Landsat 5, 7, and 8. During satellite image pre-processing, the image data were first sorted from December to February of a predetermined year and the year before and after to identify pixels with cloud coverage below 10%. A cloud score algorithm available in the GEE was used to minimize the effects of clouds and cloud shadows. This algorithm uses a mix of brightness, temperature, and the Normalized Difference Snow Index (NDSI) to compute a simple cloud-likelihood score ranging from 0 to 100 (eq. (1)). The images were masked when the score was greater than 10. High-quality no cloud satellite image data were obtained using these selected images.

(1) $$NDSI=\text{ ​}\frac{\left(G-SWIR\right)}{\left(G+SWIR\right)}$$

where; NDSI is Normalized Difference Snow Index; G and SWIR are the green and short wave infrared band.

The Google Earth Engine provides calibrated top-of-atmosphere reference (TOA) imagery of the entire Landsat satellite image collections along with georeferenced and atmospherically adjusted real-time remote sensing data. The georeferencing accuracy of each image is stored in the output file. Consequently, the Google Earth Engine does not apply any additional geometric corrections (Kumar and Mutanga, 2018; Venkatappa et al., 2020; Vos et al., 2019; Zurqani et al., 2019).

According to field observation, information from local informants, previous studies around the study area (Tewabe and Fentahun, 2020), and image classification results in the study area, there are five LULC classes, i.e., forest, bushland, grassland, cropland, and built-up area. Based on the principles of "complete consistency" and "temporal stability" (Hu and Hu, 2019); sample points were ﬁltered from ﬁve land cover types: cropland, built-up, grassland, forest, and shrubland.

Sample training data were extracted in GEE using mean pixel values of the spectral signatures These datasets were used as a training test for classifying the land use/land cover using machine learning algorithms. The proportion of its area determined the sample size for each LULC type. Training data should be representative of the entire study area. It implies that collecting more data across the study area is better than a few large training areas. Hence, adequate training sample points were obtained by considering the area size of considering the area size of each land use land cover class type in order to address misclassification errors. This procedure was followed by adding additional sample data until high accuracy classification results were obtained. The data were split randomly into the training (70%) and testing (30%) datasets.

#### 2.2.3 Classification with random forest and accuracy assessment

An efficient classifier must be chosen to classify spatial characteristics of spectra and other variables using a small number of training samples for successful classification (Foody and Mathur, 2004; Prasad et al., 2006). Machine learning-based classiﬁers are particularly useful for identifying patterns in complex functional spatial characteristics while minimizing the problem of data dimensionality (Richards and Richards, 1999).

Random forest is one of the most widely used algorithms for classifying land cover using remote sensing data (Breiman, 2001). Some researchers carried out a crucial study on LULC classification on GEE utilizing random forest algorithms and achieved remarkable outcomes (e.g., Foody and Mathur, 2004; Luo et al., 2021; Magidi et al., 2021). Therefore, this study used the Random Forest algorithm with GEE for classiﬁcation based on ﬁltered sample points. The LULC class was performed by directly calling the ee. smileRandomForest function in the GEE. Application Programming Interface (API) only requires two variables to determine the number of classification trees, and the number of feature variables entered at node splitting (Liu et al., 2020). Random Forest consists of multiple independent and unrelated decision trees. Each decision tree is judged according to the input samples and variables and predicts the samples' category (Breiman, 2001).

Furthermore, the NDVI (Bradley and Townshend, 1994), NDWI (Gao, 1996) products were used as input data for the Random Forest classiﬁcation. Thus, the satellite images of the study site were divided into five land uses (Table 2): forest, shrubland, grassland, cropland, and built-up. In this study, most processes were completed on the GEE platform (Gorelick et al., 2017), which is a highly efﬁcient free cloud platform for processing and analyzing satellite image data (Xiong et al., 2017).

Classiﬁcation algorithms were trained on 350 training samples from each class in each year, produced by the proposed automated methodology. The number of training samples was determined using a trial and error process. The performance of a classiﬁer, often known as its accuracy, can be defined as the likelihood that it will correctly classify a random set of examples (Kohavi, 1995). To assure a “fair” assessment of a classiﬁer's generalization, the data used to train the classiﬁer must be separated from the data that is used to assess its accuracy. As a result, labelled data is often separated into training and test sets (a validation set used to “tune” the classiﬁer's parameters).

Finally, the performance of the adapted Random Forest (RF) model was evaluated using test data. Test data were obtained from extensive ﬁeld visits and visual interpretation techniques using high-resolution satellite imageries (Google Earth and Sport images), topographic maps, and data from elder people's knowledge. A total of 420 samples were taken for all land use class (proportionally) each year. As a completely different procedure prepared test samples than training samples, it was possible to carefully examine how the proposed methodology performed in generating training samples. Test samples had no involvement in the training process and were also used to assess the LULC map of the study area accurately.

The accuracy was also evaluated by comparing the areal size of the classes in the classification results to the reference data set using error matrices, which describe the reliability of the map based on the data. As a result, the error matrix was used to calculate overall accuracy, producer and user accuracy, and Kappa statistic for each year (Lillesand et al., 2015).

The overall producers' classification accuracy for 1995, 2008, and 2020 is given 85.7%, 87.9%, and 89.5%, respectively, with over kappa values of 0.81, 0.85, and 0.87 (Table 1). The accuracy statistics and kappa coefficient values are considerably higher than Congalton and Green (2019) recommended values. This revealed that the Landsat images of the study periods were successfully classified. Finally, the total area and change of every LULC class were computed to evaluate the impacts of LULC change on ESV.

**Table 1:** Accuracy assessment results.

LULC class	1995		2008		2020	
	User accuracy	Producer accuracy	User accuracy	Producer accuracy	User accuracy	Producer accuracy
Cropland	82.8	80.0	89.3	83.3	87.9	85.0
Grassland	80.7	83.3	80.6	88.5	81.8	90.0
Forest	90.0	90.0	90.7	89.0	96.1	89.1
Shrubland	82.1	83.7	84.5	89.0	82.8	87.3
Built-up	97.4	95.0	97.8	90.0	100.0	94.0
Overall accuracy	85.7	85.7	87.9	87.9	88.9	88.9
Kappa Statistics	0.82	0.81	0.86	0.85	0.86	0.86

**Table 2:** The five LULC categories, biome counterparts, and value coefficients are used for estimating ecosystem service in USD ha^−1^ yr^−1^.

LULC class	Equivalent biome	Description	ESC (USD ha^−1^ yr^−1^)
Cropland	Cropland	Cultivated farmlands. It includes perennial and annual agriculture (crops) and irrigated areas.	225.56
Grassland	Grass/rangelands	Land predominantly covered by grass which is used for grazing.	293.25^a^
Forest	Tropical forest	Areas covered with a dense canopy of trees (deciduous forests, evergreen forests, mixed forests). Plantation trees were also included in this class.	986.69^a^
Shrubland	Tropical forest	Areas covered with small scattered trees (less dense than forests) and bushes	986.69^a^
Built-up	Urban	Residential, commercial, and industrial, roads, and other urban forms.	0

Belay and Mengistu (2019).

a value coefficients based on Kindu et al. (2016).

### 2.3 Estimation of ecosystem service values (ESV)

Ecosystem services and their values have been studied since the 1970s, but the Earth's ecosystem services values are difficult to assess precisely due to a lack of a corresponding theory system and method. In recent years, ecosystem service function and value assessment have become a hotspot for study (e.g., Alcamo et al., 2005; Assefa et al., 2021; Costanza et al., 1997; Gashaw et al., 2018; Kindu et al., 2016; Solomon et al., 2019; Tolessa et al., 2017). The LULC map of the three study periods (1995, 2008, and 2020) were used to assess ESVs changes. The overall workflow used for this research to detect LULC change and estimate ESVs for the specified years and study periods is depicted in Figure 2.

There are numerous ways to estimate ecosystem service values (Adamowicz et al., 2008). The benefits transfer technique, which is based on worldwide value coefficients or adjusted value coefficients published by many other scholars, has been a widely used method, especially in data-deficient areas (e.g., Costanza et al., 1997; Gashaw et al., 2018; Kindu et al., 2016; Tolessa et al., 2017), was used to evaluate the value of ecosystem services. The local modified coefficient values (USD ha^−1^ yr^−1^) developed by Kindu et al. (2016) for the terrestrial environment were used to evaluate relative losses or gains in ESV due to LULC changes. Kindu et al. (2016) designed ESV for 11 biomes depending on the Costanza et al. (1997) method considering local Ethiopian conditions. We choose only five LULC classes as a suitable proxy for LULC types: (1) cropland for cropland, (2) tropical forest for forest and shrubland, (3) grassland for grassland, (4) urban for the built-up area (Table 2).

The tropical forest biome was not an ideal proxy for forest and shrubland types because forest in the study area contained open forests, Eucalyptus plantations, and church forests. However, considering that the services provided by tropical forest and forest/shrubland of our study site are similar, we used the biome as a close approximation. However, the built-up area value is set to zero in accordance with Kindu et al. (2016) value coefficients, which were modified based on Costanza et al. (1997) because they failed to acknowledge ecosystem services such as sequestering carbon in urban areas. The LULC classes of the study area were assigned to the relevant respective biomes (Table 3).

**Table 3:** The LULC categories and their accompanying ecosystem sub-service values (USD ha^−1^ yr^−1^) for five selected biomes.

Ecosystem services	Biome			
	Cropland^a^	Shrublands	Grassland^a^	Grasslands
PES				-
Water supply		8.00		
Food production	187.56	32.00	117.45	
Raw materials		51.24		
Genetic resources		41.00		
RES				-
Water regulation		6.00	3.00	
Climate regulation		223.00		
Disturbance regulation		5.00		
Gas regulation		13.68	7.00	
Biological control	24		23.00	
Erosion control		245.00	29.00	
Waste treatment		136.00	87.00	
SES				-
Nutrient cycling		184.40		
Pollination		7.27	25.00	
Soil formation		10.00	1.00	
Habitat/refuge		17.30		
CES				
Recreation		4.80	0.80	
Cultural		2.00		
Sum	225.56	986.69	293.25	0

a value coefficients adopted from Kindu et al. (2016).

The LULC datasets to each year were made as alternatives for quantifying the ESVs, and the accompanying area in hectares was evaluated and displayed as a raster in the GIS. The ecosystem service valuation procedure assigns each LULC type a value coefficient. The value coefficients used in for this research were from Costanza et al. (1997), and the local modified ecosystem service coefficients applied were those of Kindu et al. (2016). The total monetary value of ecosystem services for 1995, 2008, and 2020 was determined by multiplying the size of a specific LULC category by the respective modified ecosystem service value coefficients derived from weight factors of ecosystem services per hectare for every biome, as shown in Eq. (2) (Kindu et al. (2016); Gashaw et al. (2018); Temesgen et al., 2018):

(2) $$ESV=\text{ ​}\sum \left(A_{k}*VC_{k}\right)$$

where ESV is the overall estimated ecosystem service value, A_k_ is the area (ha) and VC_k_ is the value coefficient (USD ha^−1^ yr^−1^) for LULC type 'k'. ESVs were calculated for all LULC types. Furthermore, the difference between estimated values for each LULC category in 1995, 2008, and 2020 was used to determine changes in ESVs.

The percent changes in ESVs between years were computed using the following formula (eq. (3)):

(3) $$Percentage\text{ ​}ESV=\left(\frac{ESV_{\mathrm{t2}}-ESV_{\mathrm{t1}}}{ESV_{\mathrm{t1}}}\right)*100$$

where ESV_t2_ (USD ha^−1^ yr^−1^) is the estimated ecosystem service value in the most recent year, and ESV_t1_ (USD ha^−1^ yr^−1^) is the anticipated ecosystem service value in the initial year. Positive values indicate an increase in the ESVs, whereas negative values indicate a decline in the ESVs.

The values of the services offered by 17 specific ecological functions within the study site were calculated using the equation below (eq. (4)):

(4) $$ESVf=\text{ ​}\sum \left(A_{k}*VCf_{k}\right)$$

where ESV_f_ is the anticipated ESV service function 'f', A_k_ is the area (ha), and VC_fk_ is the value coefficient of the function f (USD ha^−1^ yr^−1^) for LULC class 'k'. Individual ecosystem functions were assigned a value that corresponded to the total value of ecosystem services per year.

### 2.4 Ecosystem services value sensitivity analysis

The correspondence between the LULC classes and the specified biomes was not flawless, and the coefficient values were subjected to uncertainty. Notably, settlement differs from urban biomes described by Costanza et al. (1997). The ecosystem services provided by rural settlements are particularly noticeable in the provision of organic waste materials to the neighbouring garden. In the case of heat generation, urban land use creates more significant amounts of heat that are detrimental to people's health and the environment. However, both built-up area in our analysis and the urban biome described by Costanza et al. (1997) serves as a residence for humans inhabiting land for various purposes.

Likewise, shrub/bushland is not the same as a forest because it lacks the necessary canopy cover, but it provides ecosystem services comparable to the forest as described by Costanza et al. (1997). Cropland was also approximated using values of cropland. However, cropland in our instance varies in size, soil fertility, input use, and other crucial parameters. The ESV recently proposed by de Groot et al. (2012) and Costanza et al. (2014) were not used in this study because the researchers assumed that the values used by these researchers overestimate the ecosystem functions defined and are inappropriate for the land uses identified in the study area in particular and throughout the country. For instance, in Costanza et al. (2014), recreation for urban land use is one of the ecosystem services with the highest estimated ESV (USD5749 ∗10^6^). This value overlooks the monetary ESV of rural settlement because it is not a place for recreation due to its landscape structure.

Sensitivity analysis is required to determine the level of dependence of the alteration in the ESV on the coefficient values. As a result, the standard economic elasticity concept was applied to establish whether changes in the coefficient values would cause undesirable uncertainty in unit value transfer. In the sensitivity analysis, the coefficient values was used to calculate the ESV for each of the four LULC types (forest, grassland, shrubland, and cropland) were adjusted by 50% (Cai et al., 2013; Hu et al., 2008; Li et al., 2007). This method aids in determining the robustness and credibility of our estimation of ecosystem service value. The coefficient sensitivity (CS) was determined using Eq. (5) as proposed by Kreuter et al. (2001).

(5) CS=((ESVj−ESVi ​)ESViVCjk−VCikVCi)

The ESV percentage change was determined as a result of a +50% increase in the coefficient value, and LULC class 'k', "i" and "j" represent the respective initial and adjusted values, respectively. If CS is more significant than one, the anticipated ESV is elastic to that coefficient; if CS is less than one, the anticipated ESV is inelastic. The higher the corresponding change in ESV for a relative change in the coefficient value, the more vital is the use of a reliable ecosystem value coefficient. However, sensitivity analysis is commonly used in previous investigations (e.g., Crespin and Simonetti, 2016; Kindu et al., 2016; Li et al., 2007).

## 3 Results

### 3.1 Land use land cover change

**Figure 3:**
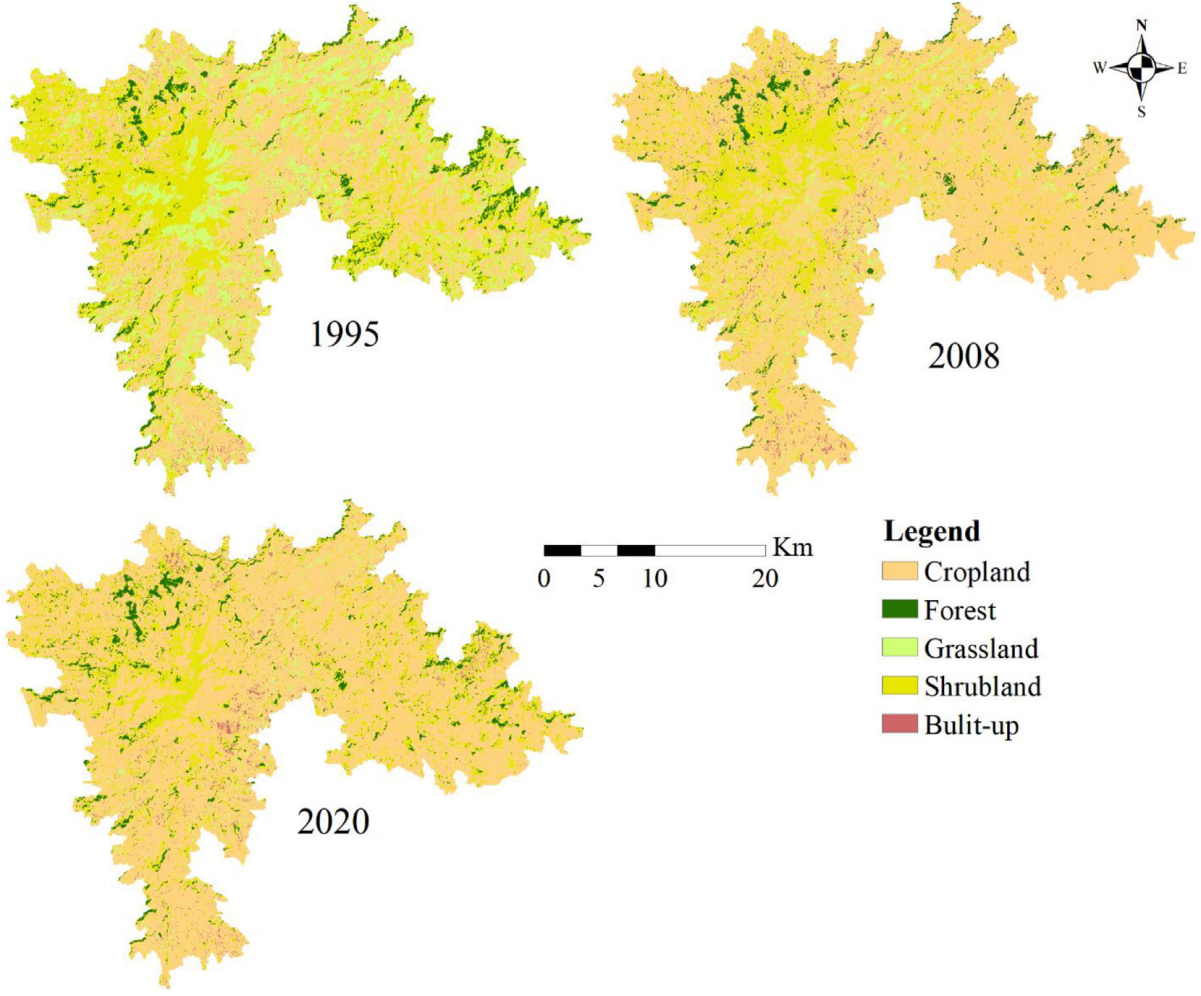
LULC classiﬁcation results for 1995, 2008, and 2020 in the Afroalpine of Guna Mountain.

The LULC maps of the study site produced for the three selected years (1995, 2008, and 2020) are presented in Figure 3. Trend analysis for two consecutive periods (1995–2008 and 2008–2020) revealed spatiotemporal changes in LULC types. The study shows that shrubland and cropland are the dominant land-use types in the study area. The LULC map of 1995 showed that 45.5% of the area was under cropland, followed by shrubland (30.0%) and grassland (19.5%). The proportion of forest and the built-up area was 4.2% and 0.6%, respectively. The finding also showed that 73.9% of the Guna mountain was covered by cropland, followed by shrubland (18.1%) and forest (4.1%). The area under built-up and grassland was estimated at 1.1% and 2.8%, respectively.

The types of LULC that showed positive changes over the analyzed 25 years were cropland and built-up areas, while forest, grassland, and shrubland showed negative changes (Table 5). The proportion of LULC types demonstrated distinct features during diﬀerent study periods. For example cropland increased by 19.4%, 2.0 %, and 61.6% during 1995–2008, 2008–2020, and 1995–2020 periods, respectively. From 1995-to 2020, the built-up area increased significantly by 85.9%. On the other hand, a tremendous decrease in the area under grassland was observed (1995–2008:10.4%), (2008–2020: 46.7%), and (1995–2020: 85.8%). Shrubland has also decreased by 8.6% (1995–2008), 0.5% (2008–2020) and 39.6% (1995–2020). The area coverage of forests diminished considerably between 1995 and 2008, but it increased rapidly during the second period of the study (Table 5). Between 2008 and 2020, the area under forest cover increased mainly due to improved land use and management practices (such as forest regeneration and afforestation), rehabilitation programs, and government and local community protection efforts.

The decline of forest, shrubland, and grassland, as well as the increase of cropland and the built-up area in the Afro-alpine area of Guna mountain over the study period (1995–2020), is consistent with previous LULC change studies conducted in various parts of Ethiopia (e.g., Belay and Mengistu, 2019; Hassen and Assen, 2018; Miheretu and Yimer, 2018; Tadesse et al., 2017).

The change matrix analysis presented in Table 4 shows the LULC conversion from one class to another. Cropland, grassland, and shrubland LULC types experienced the highest transition share during the study period. For example, over the last 25 years, croplands and built-up areas have increased at the cost of grassland (15,010 ha of grassland converted to cropland) and shrubland (15,650 ha of shrubland converted to cropland) (Table 5). The built-up area expanded primarily at the expense of cropland (830 ha) and grassland (110 ha).

**Table 4:** LULC change transition matrices (ha) from 1995 to 2020.

		LULC 2020						
		Forest	Built-up	Grassland	Cropland	Shrubland	Total 1995	Loss
LULC 1995	Forest	**2090**	10	0	320	1530	3950	−1860
	Built-up	0	**30**	30	470	30	560	−530
	Grassland	100	110	**1260**	15010	1940	18430	−17160
	Cropland	240	830	1050	**38350**	2720	43190	−4840
	Shrubland	1430	60	280	15650	**10880**	28300	−17420
	Total (2020)	3860	1040	2620	69810	17090	**52620**^a^	
	Gain	1770	1010	1360	31450	8930		
	Loss	−1860	−530	−17160	−4840	−17420		
	Net change	3630	1540	18520	36290	26350		

^a^ Is the sum of diagonal areas in hectare and indicates the total area which remained unchanged.

^b^ Net change = gain-loss.

**Table 5:** LULC extents for each type in ha and percent of the total area for 1995, 2009, 2020 and rate of change for the periods 1995–2008, 2008–2020, and 1995–2020 in the study area.

LULC Type	1995	%	2008		2020	%	1995–2008		2008–2020		1995–2020	
	Area (ha)		Area (ha)	%	Area (ha)		Area (ha/yr)	%	Area (ha/yr)	%	Area (ha/yr)	%
Forest	3950	4.2	2990	3.2	3860	4.1	−960	−0.7	880	29.4	-80	−2.2
Built-up	560	0.6	930	1.0	1040	1.1	370	0.3	110	12.1	480	85.9
Grassland	18430	19.5	4920	5.2	2620	2.8	−13510	−10.4	−2300	−46.7	−15800	−85.8
Cropland	43190	45.7	68410	72.5	69810	73.9	25220	19.4	1400	2.0	26620	61.6
Shrubland	28300	30.0	17180	18.2	17090	18.1	−11120	−8.6	−90	−0.5	−11210	−39.6

Over 25 years, cropland and built-up area showed a net gain, whereas grasslands, forestlands, and shrublands showed net losses (Table 4). Individual LULC class conversion undoubtedly impacts ecosystem functions, structures and differences in overall ESV. As a result, the following section describes how the ESV changes in response to LULC changes.

### 3.2 Values of ecosystem services over time from 1995 to 2020

The changes in ecosystem service values (ESVs) were evaluated based on the study area's classified LULC map (1995, 2008, and 2020). Over the last two decades, the study area has seen significant LULC changes because of human activities such as agricultural land expansion and urbanization. Consequently, LULC changes have had a considerable impact on ecosystem service values. LULC map of the study area was used to calculate the changes in the value of ecosystem services in Guna Mountain from 1995 to 2020. Table 6 shows the ESVs for each land use type and the total value for each study year. The total ESV of Guan mountain was estimated at USD 46.97 × 10^6^ in 1995, USD 36.77 × 10^6^ in 2008, and USD 37.19 × 10^6^ in 2020 (Table 6). The net ESVs of the study site declined by USD 9.78 × 10^6^ between 1995 and 2020, at a rate of 0.3912% per year. The loss of total ESV is most likely due to the conversion of forestland, shrubland, and grassland to farmland, which has a higher ESV per hectare.

**Table 6:** The overall estimated ecosystem service values (USD x 10^6^) in the study area.

LULC Type	ESV						ESVs change					
	1995		2008		2020		1995–2008		2008–2020		1995–2020	
	ESV	%	ESV	%	ESV	%	ESV	%	ESV	%	ESV	%
Forest	3.90	8.30	2.95	8.02	3.81	10.24	−0.95	−24.30	0.86	29.10	−0.09	−2.28
Built-up	0.00	0.00	0.00	0.00	0.00	0.00	0.00	0.00	0.00	0.00	0.00	0.00
Grassland	5.40	11.51	1.44	3.92	0.77	2.07	−3.96	−73.30	−0.67	−46.75	−4.64	−85.78
Cropland	9.74	20.74	15.43	41.96	15.75	42.35	5.69	58.39	0.32	2.05	6.00	61.63
Shrubland	27.92	59.45	16.95	46.09	16.86	45.35	−10.97	−39.29	−0.09	−0.52	−11.06	−39.61
Sum	46.97	100	36.77	100	37.19	100	−10.19	−21.71	0.42	1.14	−9.78	−20.82

The monetary value of ecosystem services supplied by various land types varied. From 1995-to 2020, the ESV of the forest declined, whereas the ESV of croplands increased. The study indicated that the ESVs of cropland and forest were higher in 2020 than in 1995. The ESVs of grassland and shrubland declined by USD 4.64 × 10^6^ and x USD 10^6^, respectively. The ESV reduction was more remarkable in grassland (85.78%), followed by shrubland (39.61%), as shown in Table 6. This is most likely due to a considerable decrease in the areal extent of grassland and shrubland. The ESVs of forestland decreased slightly, by USD 0.09 × 10^6^ (Table 6).

In contrast, the ESV of cropland increased by USD 61.63 × 10^6^ between 1995 and 2020. Each studied period saw a decrease in ESV for grassland and shrubland. The highest ecosystem service value for the year 1995 was USD 27.92 × 10^6^ from shrublands, followed by croplands with USD 9.74 × 10^6^. Nevertheless, the contribution of ESV from shrubland decreased while the contribution from cropland increased in 2020.

### 3.3 Impacts of LULC changes on distinct ecosystem service values

The ecosystem service values provided by separate ecosystem services are shown in Table 7. The table shows that LULC changes have various effects on the different ecosystem services in the study area. The value of regulating services held the greatest share, accounting for more than 42% across all periods, followed by provisioning and supporting services, which attributed for more than 29% and 13%, respectively. In contrast, cultural services contributed the least to the total ESV. Regulating services showed a downward tendency throughout all periods while supporting services showed an initial decreasing trend followed by an upward trend.

**Table 7:** LULC change and its impact on ecosystem service values.

Ecosystem Services		ESVs across periods (USD in millions)			Overall change
Groups	Indicators	ESVf 1995	EStainVf 2008	ESVf 2020	1995–2020
PES	Water supply	0.35	0.25	0.26	−0.09
	Food production	11.39	14.14	14.16	2.77
	Raw materials	1.75	1.12	1.16	−0.59
	Genetic resources	0.42	0.34	0.41	−0.01
	Total	13.91	15.85	15.99	2.08
RES	Water regulation	0.34	0.23	0.22	−0.12
	Climate regulation	7.29	4.59	4.76	−2.53
	Disturbance regulation	0.25	0.19	0.19	−0.06
	Gas regulation	0.66	0.40	0.39	−0.27
	Biological control	1.55	1.85	1.82	0.27
	Erosion control	8.53	5.17	5.30	−3.23
	Waste treatment	6.08	3.26	3.17	−2.91
	Total	18.62	12.43	12.68	−5.94
SES	Nutrient cycling	6.04	3.81	3.95	−2.09
	Pollination	0.80	0.36	0.31	−0.49
	Soil formation	0.43	0.30	0.30	−0.13
	Habitat/refuge	0.65	0.44	0.45	−0.2
	Total	7.92	4.91	5.01	−2.91
CES	Recreation	0.26	0.19	0.19	−0.07
	Cultural	0.16	0.13	0.13	−0.03
	Total	0.42	0.32	0.32	−0.1

Note: PES -provisioning, RES-regulatory, SES-supporting, and CES-cultural ES.

Provisioning ecosystem services had increasing trends from 1995 to 2020, which increased slightly by USD 2.08 × 10^6^. The increase in cropland provisioning service has been caused by increased cropland size and a greater ecosystem service value attributed to it. On the other hand, regulating services, supporting services, and cultural services between 1995 and 2020 were decreased by USD 5.94 × 10^6^, USD 2.91 × 10^6^, and USD 0.1 × 10^6^, respectively. The individual ESV function (ESV_f_) results showed a decreasing trend for each period study. In terms of the ESV_f_, food production contributed most to the total ESV, followed by erosion control, climate regulation, nutrient cycling, waste treatment, biological control, and raw materials. From the listed ecosystem, service indicators, cultural and disturbance regulation services contributed the least in 1995, 2008, and 2020.

The ecosystem service value of food production experienced the highest increase, about USD 2.77 × 10^6^, which is most likely related to the expansion of cropland. However, the ecosystem service value of the remaining 16 individual ecosystem service values declined with varying degrees of reduction from 1995 to 2008 and 2008 to 2020 (Table 7). The highest ecosystem service losses were found in erosion control (USD 3.23 × 10^6^), followed by waste treatment (USD 2.91 × 10^6^), climate regulation (USD 2.53 × 10^6^), and nutrient cycling (USD 2.09 × 10^6^) (Table 7). From 1995 to 2020, the total ESV of the study site declined by US $9.78 × 10^6^. The loss of shrubland and grassland has reduced the value of ecosystem services, which resulted in losses of USD 11.06 × 10^6^ and USD 4.64 × 10^6^, respectively. Therefore, it is critical to maintain the integrity and area of natural habitats in the study area to sustain various ecosystem benefits and services and their long-term flow in the study site.

## 4 Discussion

Land use and land cover change (LULCC) is unavoidable due to socio-economic development (Wu et al., 2008). In this regard, Ethiopia's recent studies on LULC change revealed considerable land-use changes. According to this study, in the last 25 years, there has been a massive LULC change, mainly in built-up, farmland, shrubland, and grassland areas. Similarly, studies on LULC change in Ethiopia's highlands have been carried out (e.g., Belay and Mengistu, 2019; Hassen and Assen, 2018, 2018; Tadesse et al., 2017), and they also showed the expansion of cropland was increased at the expense of forest and grassland. Cropland expansion was exacerbated by the rapid growth of the rural human population. Subsistence agriculture is the main source of income for the rural community. The local community of the study area illegally converts forest, grassland, and shrubland areas into farmland (Birhanu et al., 2021). Cropland expansion without adequate land management will result in the loss of ecological services. In addition, road construction exacerbated forest fragmentation, allowing for the expansion of agricultural lands and settlements. Natural habitat in the area has deteriorated as a result of increased LULC caused by human activities.

The effects of LUCC change on ecosystem services are highly complicated. LULCC cause ecosystem structure and function and ESV changes per unit area (Gashaw et al., 2018; Solomon et al., 2019; Temesgen et al., 2018). Some researchers have looked into the impacts of LULCC on the ESV in Ethiopia. They pointed out that the loss of forest, shrubland, and grasslands in the Abay river basin was the primary cause of ESV reduction.

The most common LULC change in the study area was from shrubland to cropland, followed by grassland. The loss of ESVs was associated with the conversion of vast amounts of shrubland and grassland to forest. The loss of forestland also led to the decline of ESVs. The transformation in LULC from grassland, shrubland, and forest has resulted in a significant decrease in raw material production in Guna mountain, which is consistent with previous studies conducted in different parts of Ethiopia (Gashaw et al., 2018; Solomon et al., 2019; Tolessa et al., 2017). In addition, the significant decline in several ES functions has vital policy implications for balancing food security and ecological services. This is consistent with the findings of previous studies, which show that providing various ecosystem services, for instance, food production (Rimal et al., 2019), raw materials (Arowolo et al., 2018; Tolessa et al., 2017), waste treatment (Hasan et al., 2020), and soil formation (Arowolo et al., 2018; Badamfirooz and Mousazadeh, 2019), is detrimental to agricultural reduction and urban expansion. Overall, the findings of this study highlight the importance of natural ecosystems in providing essential ecosystem services for human well-being (Costanza et al., 2017; Keesstra et al., 2018). Furthermore, this research could contribute to a better understanding of how changes in LULC affect ecosystem service values provided by the landscape reserve.

This study's findings are consistent with previous research in Ethiopia (Kindu et al., 2016; Tolessa et al., 2018; Weldegebriel and Yeshitela, 2021) and elsewhere (e.g., Aziz, 2021; Hoque et al., 2020; Huq et al., 2019; Sun et al., 2021; Zhang et al., 2017, 2019). According to the findings of these studies, LULC dynamics impact the overall ESV at the landscape level.

### 4.1 Implications for landscape management

Economic progress and social prosperity necessitate LULC change (Wu et al., 2008), but it frequently derives high environmental costs. The study area's ecological services have been severely impacted by the rapid increase of cropland and built-up areas at the expense of forest, grassland, and shrubland.

The conversion of grassland and forest to cropland and built-up area is expected to have an impact on the hydrological cycle, increasing soil erosion and runoff. This, in turn, leads to the deterioration of ecological services, which may have an impact on the local community's livelihood. Proper grassland, forest, and shrub-bushland management is critical for the long-term viability of Guna mountain's resources. Appropriate grassland, forest, and shrub-bushland management are essential for the long-term viability of Guna Mountain's resources.

The focus of this study was to examine the relationship between spatiotemporal LULC dynamics and total ESV changes. Birhanu et al. (2021) discovered that recent development activities and land-use-related policy caused accelerated LULC changes, resulting in a decline and loss of ESV in a similar study area. Thus, the study on ESV changes in LULC patterns produced valuable evidence to support policymakers in various ways. The findings of this study could be used to develop appropriate management strategies, highlighting the possible effects of land-use dynamics on ESV at different temporal and spatial scales. The results will be used to contrast and highlight the rate of change in ES and its accompanying values. Despite the known sources of constraint associated with transferring values as a rough approximation (Kindu et al., 2016), the research findings can serve as a springboard for future scientific inquiry in this circumstance. The study could serve as a foundation for policymakers to change their land-use policies. Furthermore, the study's findings contribute to developing appropriate natural resource management approaches that assure long-term use and management of natural resources.

A study that substantiated the ecosystem service loss in terms of money could help higher-level officials comprehend the implications of LULC changes on the total ES and its function. The study concluded that practicing conservation land management strategies like crop residue management, conservation agriculture (minimum tillage, crop rotation, permanent cover crops, intercropping, etc.), agroforestry practices, and water harvesting, which are important for improving ecosystem services without compromising agricultural products, are critical. Grassland and shrubland conversion to ecological lands is vital.

### 4.2 Limitations of the study

The impacts of LULC changes in ESV for the period 1995–2020 incorporate various sources of uncertainty. The study was based on LULC, which was limited by the resolution of remote sensing images and the limitation of algorithms, resulting in some classification errors. Besides, the ESV assessed in 2020 was summarized to the average unit value of all different periods, which did not consider the changing crop prices over time. As a result, ESV was computed because the value of ecosystem services for the same land type was uniform over the years, and thus the ESV estimated was constant.

## 5 Conclusion

This research focuses on the impacts of LUCC changes on ecosystem service values in the Afro-alpine area of Guna mountain. Cropland was the primary land use type in the study area between 1995 and 2020, and its proportion indicated an increasing trend. Forest and built-up areas accounted for a small proportion of the total, with the built-up area increasing slowly over time. In contrast, the share of forests declined during the second period but increased during the second period. The areas of built-up land and cropland increased continuously. From 1995 to 2020, the estimated total ecosystem service values of Guna mountain decreased significantly by $UD 9.78 × 10^6^, which was a decline of 20.82%. The study results proved the effect of changes in LULC on ecosystem services. It can be used as a scientific and practical reference program to protect and conserve afro-alpine mountainous areas and give scientific support for mountain area restoration.

According to this study, there has been a significant increase in cropland and settlement areas, mainly at the expense of natural habitats, resulting in decreases in total and individual ESV over the studied periods. The most important contributing factor to the reduction of the study area's total ESV was a significant decline in grassland and shrubland over the study periods. Due to land-use dynamics, the quantity of ESV lost was more significant than that of ESV acquired. Individual ESV from the research location decreased from 1995 to 2020 due to the LULC modification. As a result, reversing the trend of increasing the area size of natural habitats such as forest, grassland, and shrubland is critical to maintaining and protecting the ecosystem service values of Guna mountain.

The findings of this study will be critical in determining and executing appropriate development policies that promote agricultural land expansion to boost vegetative cover and reduce ESV loss in the study area. Similarly, the findings of this research can be used by local and regional government agencies, developers, and policymakers to prevent ESV degradation in the Guna mountain's Afro-alpine area.

## Declarations

### Author contribution statement

Tatek Belay: Conceived and designed the experiments; Performed the experiments; Analyzed and interpreted the data; Wrote the paper.

Tadele Melese; Abebe Senamaw: Analyzed and interpreted the data; Contributed reagents, materials, analysis tools or data.

### Funding statement

This research did not receive any specific grant from funding agencies in the public, commercial, or not-for-profit sectors.

### Data availability statement

Data will be made available on request.

### Declaration of interest's statement

The authors declare no competing interests.

### Additional information

No additional information is available for this paper.
